# Peripheral nerve blocks for total knee arthroplasty: a bibliometric and visual analysis, 2016–2025

**DOI:** 10.3389/fmed.2026.1903949

**Published:** 2026-08-17

**Authors:** Hao-Tian Li, Guang-Yong Lin, Yu Liu, Chuan-Xiang Zhang

**Affiliations:** 1 Department of Anesthesiology, Wuxi Xinwu District Xinrui Hospital, Wuxi, Jiangsu, China; 2 Department of Anesthesiology, The Third People’s Hospital of Longgang, Clinical Institute of Shantou University Medical College (The Third People's Hospital of Longgang District Shenzhen), Shenzhen, Guangdong, China

**Keywords:** adductor canal block, bibliometric analysis, IPACK block, liposomal bupivacaine, peripheral nerve block, total knee arthroplasty

## Abstract

**Background:**

Total knee arthroplasty (TKA) is associated with substantial postoperative pain that may delay mobilization and recovery. Peripheral nerve blocks (PNBs) are widely studied within multimodal analgesia and enhanced recovery after surgery (ERAS) pathways, but the structure and evolution of this literature have not been comprehensively mapped. This bibliometric study evaluated global publication trends, collaboration patterns, citation structures, and prominent research themes in PNB research for TKA from 2016 to 2025.

**Methods:**

Records published from January 1, 2016, to December 31, 2025, were retrieved from the Web of Science Core Collection (WoSCC) and Scopus on January 7, 2026. After database-specific filtering, multistep deduplication, and eligibility screening, 1,225 unique English-language articles were included. Bibliometric analyses were conducted using VOSviewer (version 1.6.20), CiteSpace (version 6.2.R4), and the bibliometrix package in R (version 4.1.2). Full database-specific strategies, screening criteria, data harmonization rules, and analysis settings are provided in the Supplementary Methods.

**Results:**

Annual publication output fluctuated but increased overall from 105 records in 2016 to 129 in 2025, with a peak of 134 in 2024. The compound annual growth rate was 2.31% across the nine year-to-year intervals from 2016 to 2025. The United States and China contributed the largest publication volumes, and the Hospital for Special Surgery was the most productive institution. Keyword clustering identified nine clusters (modularity *Q* > 0.7; mean silhouette >0.8), demonstrating substantial publication activity around motor-sparing techniques, including adductor canal block and IPACK, as well as liposomal bupivacaine and chronic postsurgical pain.

**Conclusion:**

The literature on PNBs for TKA has increasingly examined motor-sparing techniques, functional recovery, long-acting local anesthetic formulations, and longer-term outcomes. Liposomal bupivacaine, chronic postsurgical pain, sleep disturbance, and cognitive outcomes were prominent themes. However, bibliometric prominence should not be interpreted as evidence of clinical superiority or as a basis for treatment recommendations. These findings identify areas of research activity and uncertainty that warrant further evaluation through comparative clinical studies and systematic evidence synthesis.

## 1 Introduction

Total knee arthroplasty (TKA) is a well-established surgical procedure for end-stage knee osteoarthritis that offers substantial improvements in pain, joint function, and health-related quality of life (1). As the global population ages and obesity rates increase, the demand for TKA is projected to increase significantly in the coming decades (2). However, despite advances in surgical techniques and implant design, postoperative pain remains a major challenge and barrier to recovery after TKA.

The origin of pain after TKA is multifactorial, stemming from bone resection, soft tissue trauma, and the postoperative inflammatory response (3). Inadequate control of acute postoperative pain can delay rehabilitation, prolong hospitalization, and impair functional recovery. Notably, poor acute pain management has been associated with an increased risk of chronic postsurgical pain (CPSP), which affects approximately 20% of patients after knee replacement (4, 5). Persistent pain is linked to worse functional outcomes, lower quality of life, and increased healthcare resource utilization.

Traditionally, pain management after TKA relies on systemic opioids and neuraxial techniques such as epidural analgesia (1). Although these methods provide effective pain relief, they are frequently associated with adverse effects, including nausea, hypotension, urinary retention, and opioid-related complications. Given the ongoing opioid crisis, there has been a growing shift toward opioid-sparing strategies, with multimodal analgesia now becoming the standard for postoperative pain management.

Among multimodal analgesic strategies, peripheral nerve blocks (PNBs) have been widely investigated for their potential to reduce pain and opioid consumption. Femoral nerve block (FNB) has historically been used after TKA; however, its association with quadriceps weakness may limit early mobilization and recovery (3, 6). Adductor canal block (ACB) has therefore been studied as a motor-sparing alternative that may provide similar analgesia with less impairment of quadriceps function (6, 7). More recently, infiltration between the popliteal artery and the capsule of the knee (IPACK) block has been investigated as an adjunct for posterior knee pain while aiming to preserve motor function (8).

Although peripheral nerve block techniques have been extensively investigated, previous evidence syntheses have reached heterogeneous conclusions regarding the optimal analgesic strategy for TKA. Bibliometric analysis complements conventional evidence synthesis by providing a field-level perspective on publication growth, collaborative relationships, citation structures, and thematic development (9). Because bibliometric prominence does not necessarily reflect clinical effectiveness or evidence quality, clinical interpretations in this study were informed by relevant trials, systematic reviews, meta-analyses, and guidelines rather than inferred solely from publication or citation patterns.

Despite the expanding literature, research on PNBs for TKA remains distributed across diverse block techniques, outcome domains, and geographic settings. In particular, the emergence and relative prominence of motor-sparing approaches, including ACB and IPACK, have not been comprehensively characterized using an integrated multidatabase bibliometric framework. Therefore, this study aimed to map the global development of original research on PNBs for TKA from 2016 to 2025 by examining publication trends, collaboration networks, citation relationships, and evolving research themes. By delineating the intellectual and collaborative structure of the field, we sought to identify underexplored areas and opportunities for future investigation rather than establish the clinical superiority of any specific block technique.

## 2 Methods

### 2.1 Data sources

We used the Web of Science Core Collection (WoSCC) and Scopus as the two primary data sources. The searches covered records with publication years from January 1, 2016, through December 31, 2025. The WoSCC search was performed on January 7, 2026, and the Scopus search was performed on January 7, 2026. No records retrieved after these search dates were added to the dataset.

The complete database-specific strategies are provided in Supplementary Table S1. The WoSCC query was: TS=((“total knee arthroplasty” OR TKA OR “total knee replacement”) AND (“peripheral nerve block*” OR “adductor canal block*” OR “femoral nerve block*” OR IPACK OR iPACK OR “local infiltration analgesia”)) AND PY=(2016–2025) AND LA=(English) AND DT=(Article). The Scopus query was: TITLE-ABS-KEY((“total knee arthroplasty” OR TKA OR “total knee replacement”) AND (“peripheral nerve block*” OR “adductor canal block*” OR “femoral nerve block*” OR IPACK OR iPACK OR “local infiltration analgesia”)) AND PUBYEAR > 2015 AND PUBYEAR < 2026 AND LIMIT-TO(LANGUAGE,"English”) AND (LIMIT-TO(DOCTYPE,"ar”)).

The WoSCC Topic field searches titles, abstracts, author keywords, and Keyword Plus, whereas Scopus TITLE-ABS-KEY searches titles, abstracts, and author/indexed keywords. Full terms, acronyms, wildcards, and spelling or capitalization variants, including IPACK and iPACK, were considered. Only English-language articles were eligible. Conference papers or proceedings, meeting abstracts, editorials, letters, notes, corrections, book chapters, reviews, and other non-article publication types were excluded. Early-access, online-first, and article-in-press records classified as Articles were eligible. When both a preliminary and final version of the same publication were identified, the records were consolidated through DOI- and metadata-based matching, and the final publication metadata, including volume, issue, and pagination or article number, were retained. A unique preliminary record without a matching final version was retained as a single publication.

All retrieved records were independently screened by two researchers using predefined eligibility criteria. Records were eligible when they: (1) concerned patients undergoing TKA or total knee replacement, or reported a separable TKA subgroup; (2) evaluated, compared, or described at least one named peripheral nerve block; (3) were indexed as an Article; (4) were published in English; and (5) had a publication year from 2016 through 2025. Records were excluded when they concerned non-TKA surgery without extractable TKA data, addressed only systemic or neuraxial analgesia, were ineligible publication types, or lacked a peripheral nerve block component. Studies of local infiltration analgesia were retained only when a named peripheral nerve block was evaluated in the same study or comparative framework. Periarticular, intra-articular, or wound infiltration alone, without a nerve-targeted injection or named peripheral nerve block, was classified as local-infiltration-only and excluded. Detailed eligibility rules are provided in Supplementary Table S2.

**Figure 1:**
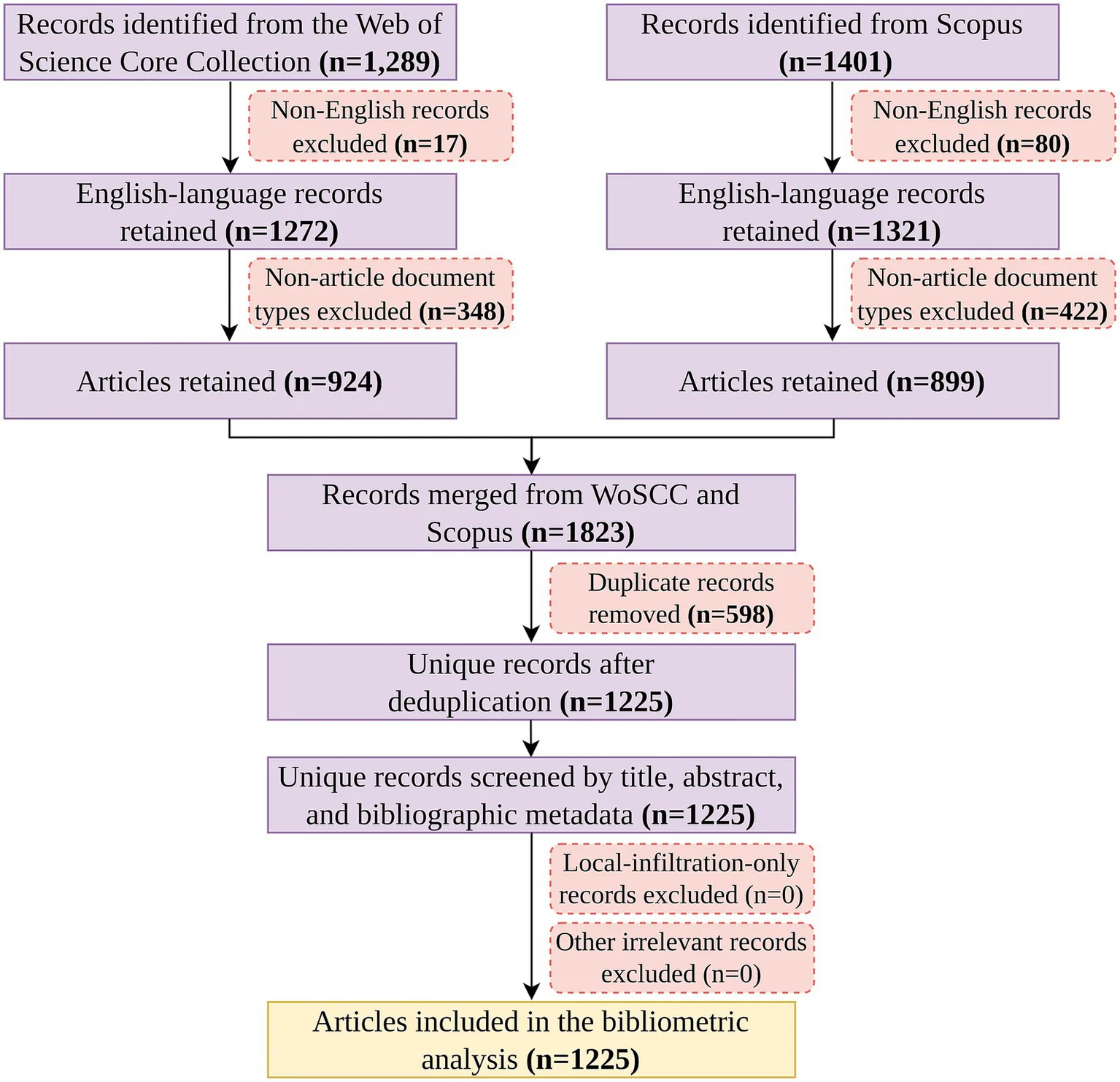
Literature identification, deduplication, screening, and inclusion process. Records were retrieved from the WoSCC and Scopus. After restricting the results to English-language articles, the database records were merged and deduplicated using hierarchical DOI and title-based matching. The remaining unique records were screened using titles, abstracts, and bibliographic metadata to confirm the presence of a peripheral nerve block component and to exclude studies focused exclusively on local infiltration analgesia or other irrelevant topics. A total of 1,225 articles were included in the final bibliometric analysis. Detailed stage counts are provided in Supplementary Table S5.

Screening was based on titles, abstracts, document types, and available metadata; full text was consulted when relevance could not be determined from metadata alone. Disagreements were resolved by consensus. The initial searches yielded 1,289 WoSCC records and 1,401 Scopus records. After the English-language and Article filters, 924 WoSCC and 899 Scopus records remained. The combined 1,823 records were deduplicated, with 598 duplicate records removed, yielding 1,225 unique publications. The complete reconciliation is reported in Figure 1, Supplementary Table S5.

### 2.2 Data integration and preprocessing

Bibliographic records were exported from WoSCC in plain-text format and from Scopus in CSV format, including titles, abstracts, authors, affiliations, author keywords, source information, cited references, DOI, publication year, and database-specific citation counts when available. DOIs were normalized by lowercasing and removing URL or prefix variants. Deduplication followed a hierarchical procedure: (1) exact DOI matching; (2) exact matching of normalized titles; and (3) matching of normalized title, first author, publication year, and source for records with missing or discordant DOIs. Title normalization included lowercasing, punctuation removal, whitespace standardization, and removal of non-informative formatting.

#### 2.2.1 Data integration and deduplication

Duplicate records were identified using a hierarchical procedure. Exact DOI matches were removed first. For records with missing or discordant DOI information, titles were normalized by converting text to lowercase and standardizing punctuation, spacing, and non-informative characters. Fuzzy title matching was subsequently performed using the duplicated Matching function in the bibliometrix R package (Field = “TI,” exact = FALSE, tol = 0.95), which applies a restricted Damerau–Levenshtein similarity measure. Candidate matches generated during fuzzy matching were manually reviewed by two investigators, and disagreements were resolved through discussion and consensus.

For confirmed cross-database duplicates, a single master record was retained according to a prespecified source-priority rule. The WoSCC record was selected when the publication was indexed in both databases, whereas the Scopus record was retained for publications uniquely retrieved from Scopus. Non-conflicting bibliographic fields were supplemented from the corresponding duplicate record when necessary, whereas conflicting fields followed the selected master source. Database-specific citation counts were neither summed nor averaged. Missing metadata were retained as missing and were not imputed. The final integrated dataset comprised 1,225 unique publications. Detailed rules are provided in Supplementary Methods S3.

#### 2.2.2 Author name disambiguation

Author names were disambiguated using a combination of automated normalization and manual verification. Punctuation, spacing, capitalization, initials, and name-order variants were standardized. ORCID identifiers, full author names, affiliations, co-author networks, and publication topics were considered when available. High-output authors and potentially ambiguous name variants were manually examined. Author entities were not merged when the available metadata were insufficient to establish that they represented the same individual.

#### 2.2.3 Institutional normalization

Institutional affiliations were standardized using a predefined thesaurus. Abbreviations, alternative English translations, department-level variants, historical name changes, and institutional mergers were manually harmonized. Department-level affiliations were consolidated at the institutional level. Affiliated hospitals were assigned to their formally affiliated parent universities according to a prespecified parent-institution rule, and publications from multiple hospitals affiliated with the same university were aggregated under the corresponding university name. Independent hospitals and medical centers without an identifiable parent university were retained as separate institutional entities. When an affiliation contained both a hospital and its parent university, the university was retained as the normalized institutional entity.

Country/region names and keywords were standardized separately using prespecified thesauri. Alternative country/region names and database-specific variants were converted to preferred forms. Keyword variants arising from capitalization, spelling, British and American English, hyphenation, singular and plural forms, abbreviations, and closely equivalent expressions were consolidated under preferred terms. These procedures were distinct from author-name disambiguation. Detailed normalization rules are provided in Supplementary Methods S4.1.

#### 2.2.4 Citation analysis and self-citations

Database-derived citation-count indicators and citation-based publication rankings were restricted to records indexed in the WoSCC. The WoSCC Times Cited value was used as the prespecified and uniform citation-count source. Publications uniquely retrieved from Scopus were retained in the integrated dataset for analyses of publication trends, authorship, institutions, countries/regions, collaboration networks, and keywords, but were excluded from indicators and rankings requiring a directly comparable database-derived citation count. Citation counts from WoSCC and Scopus were neither summed nor averaged.

The unadjusted WoSCC Times Cited values were used in the primary analysis, and author self-citations were not removed. A sensitivity analysis excluding author self-citations was not performed because sufficiently reliable identification would have required consistent matching of author identities across all citing and cited records, which could not be ensured because of residual variation in author names and affiliations. The possible inflation of citation-based rankings and impact indicators by self-citation is therefore acknowledged as a limitation. Citation counts were interpreted as indicators of database-recorded scholarly visibility rather than direct measures of methodological quality or clinical importance.

### 2.3 Analysis methods

Bibliometric analysis and visualization were performed using CiteSpace (version 6.2.R4), VOSviewer (version 1.6.20), and the bibliometrix package in R (version 4.1.2). CiteSpace was used to detect reference citation bursts and generate keyword-clustering maps using the log-likelihood ratio algorithm; principal settings included 1-year time slices and a minimum cluster size of 20 nodes. VOSviewer was used to construct author, institution, country/region, journal co-citation, and keyword co-occurrence networks. Bibliometrix was used to summarize annual publication output, authorship characteristics, collaboration indicators, and citation-based metrics.

Full counting was used for the author, institution, and country/region analyses. Each distinct author, normalized institution, and country/region received one full publication credit per document, with repeated occurrences of the same entity within a single publication counted only once. Authors with multiple affiliations were counted once in the author analysis, whereas all distinct normalized affiliations were retained in the institution analysis. Affiliated hospitals were assigned to their parent universities according to the predefined institutional thesaurus, and independent hospitals without an identifiable parent university were retained separately. Networks were normalized using the association-strength method. The minimum inclusion thresholds were 2, 3, and 5 publications for authors, institutions, and countries/regions, respectively. The country/region chord diagram displayed the 20 most productive countries/regions. VOSviewer’s label-overlap reduction was applied for readability and affected visualization only. Full analytical parameters are reported in Supplementary Methods S4.2.

The compound annual growth rate was calculated as CAGR = [(N2025/N2016)^(1/9) − 1] × 100, because 2016–2025 contains nine year-to-year intervals. A descriptive three-year moving average may be shown to reduce annual fluctuation. The cumulative publication curve and its linear R^2^ were not used as evidence of stable expansion because cumulative data are inherently monotonic.

### 2.4 Interpretive scope of the bibliometric analysis

Bibliometric indicators were used to characterize publication activity, scholarly visibility, collaboration patterns, citation structures, and keyword relationships. They do not directly assess comparative effectiveness, safety, risk of bias, evidence certainty, or clinical superiority. Keyword prominence, cluster size, and citation bursts were therefore interpreted as indicators of research activity and thematic attention rather than evidence strength or clinical importance. Clinical interpretations were supported by independently cited trials, systematic reviews, meta-analyses, or guidelines and were not inferred solely from bibliometric prominence.

## 3 Results

### 3.1 Basic information and publication trends

**Figure 2:**
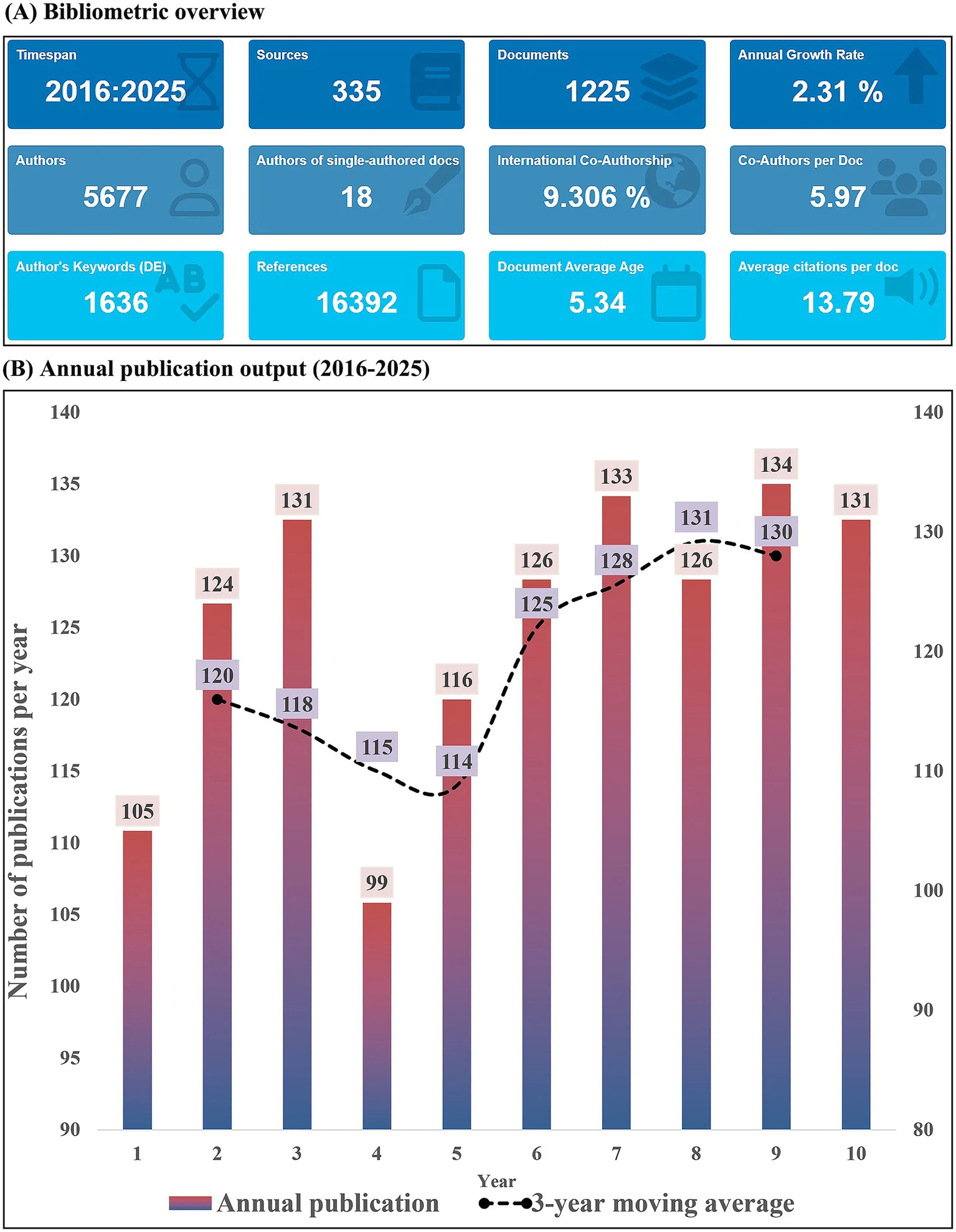
Overview of publication characteristics and annual trends. **(A)** Summary of bibliometric indicators. **(B)** Annual publication counts from 2016 to 2025 with a descriptive 3-year moving average. The CAGR was calculated from the first and final annual counts over nine year-to-year intervals. CAGR = [(*N*2025 / *N*2016)^(1/9) − 1] × 100 = 2.31%. The moving average is descriptive.

The final analysis included 1,225 documents published between 2016 and 2025 across 335 sources (Figure 2A). A total of 5,677 authors contributed to the field, whereas 18 documents were single-authored, indicating a highly collaborative publication pattern. The mean number of co-authors per document was 5.97.

Annual publication output fluctuated across the study period but increased overall from 105 publications in 2016 to 129 in 2025, with a peak of 134 in 2024 (Figure 2B). The compound annual growth rate was calculated as CAGR = [(N2025/N2016)^(1/9) − 1] × 100 and was 2.31%. The cumulative publication curve and its linear *R*^2^ were not interpreted because cumulative series are inherently monotonic and may produce spuriously high goodness-of-fit values. Annual counts and a descriptive three-year moving average provide a more informative summary of publication activity.

### 3.2 Analysis of authors and journals

#### 3.2.1 Author productivity and collaboration

**Figure 3:**
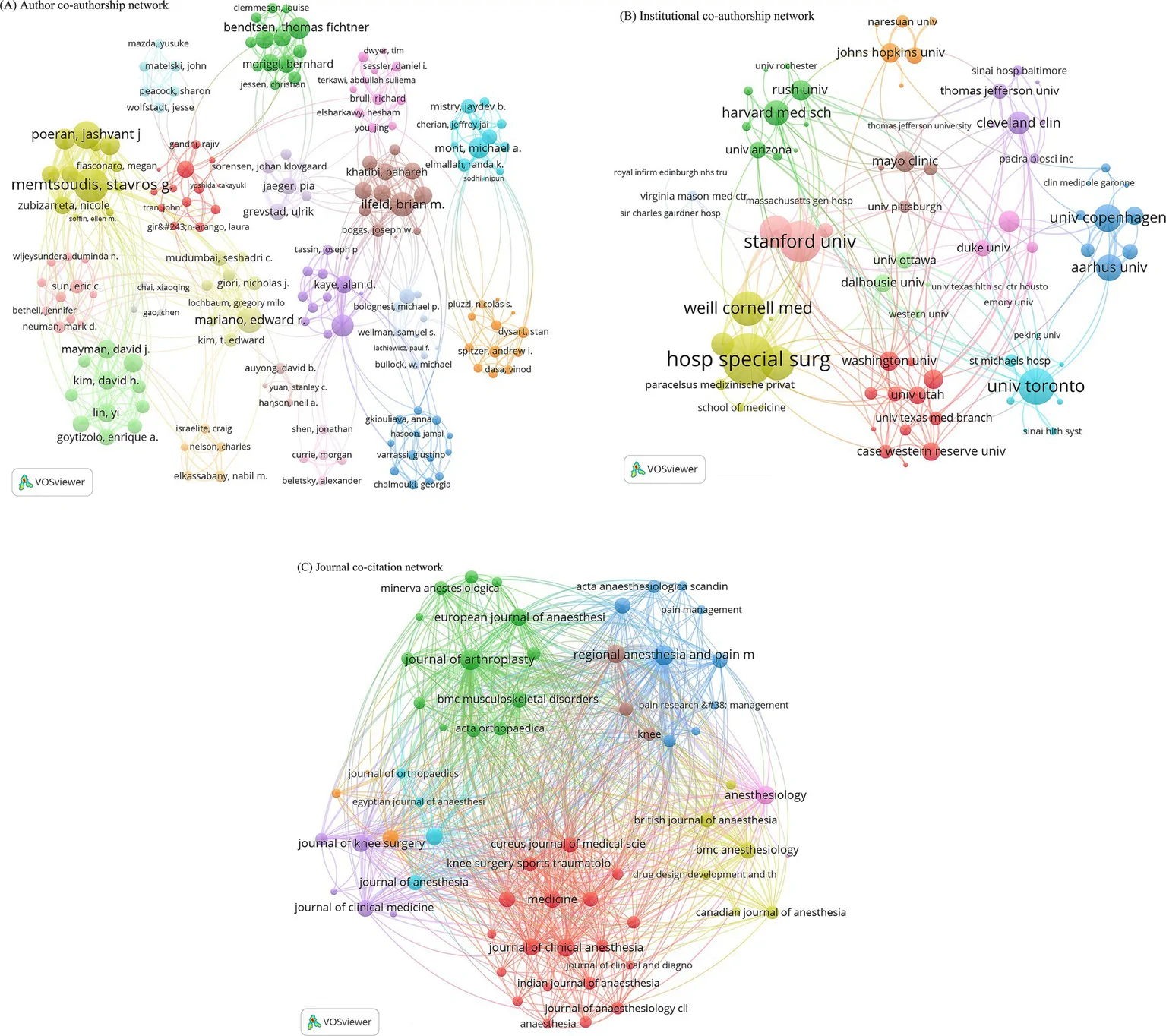
Network visualizations of authors, institutions, and journals in peripheral nerve block research for total knee arthroplasty. **(A)** Author co-authorship network. Authors with at least two publications were included in the analysis (*n* = 188). Full counting was used, with no minimum citation threshold applied. Node size is proportional to publication output, link width represents co-authorship strength, and node color indicates cluster membership identified by VOSviewer. **(B)** Institutional co-authorship network. Institutions with at least three publications were included after institutional-name normalization (*n* = 86). Full counting was used, with no minimum citation threshold applied. Node size reflects institutional publication output, link width indicates interinstitutional co-authorship strength, and node color represents cluster membership. **(C)** Journal co-citation network. Journals cited at least five times were included (*n* = 65). Node size is proportional to journal citation frequency, link width represents co-citation strength between journals, and node color indicates cluster membership. The three panels were exported separately at enlarged dimensions to improve label readability. Enlargement altered only the visual presentation and did not modify the included nodes, links, weights, or cluster assignments.

The author collaboration network (Figure 3A) showed several prominent clusters. Memtsoudis occupied a central position and connected multiple collaborative groups; Edward R. Mariano and Brian M. Ilfeld were also among the most productive and well-connected authors. These patterns describe network position and collaboration intensity within the indexed literature and should not be interpreted as direct measures of research quality.

#### 3.2.2 Core journals

A total of 335 journals published studies on PNBs in TKA (Figure 3C). The Journal of Arthroplasty and Regional Anesthesia & Pain Medicine ranked highly in publication volume and co-citation frequency. Other visible journals included The Journal of Bone and Joint Surgery and Anesthesia & Analgesia. These co-citation patterns indicate intellectual connections between orthopedic and anesthesiology literature but do not directly measure the methodological quality of the cited studies.

### 3.3 Analysis of countries/regions and institutions

**Figure 4:**
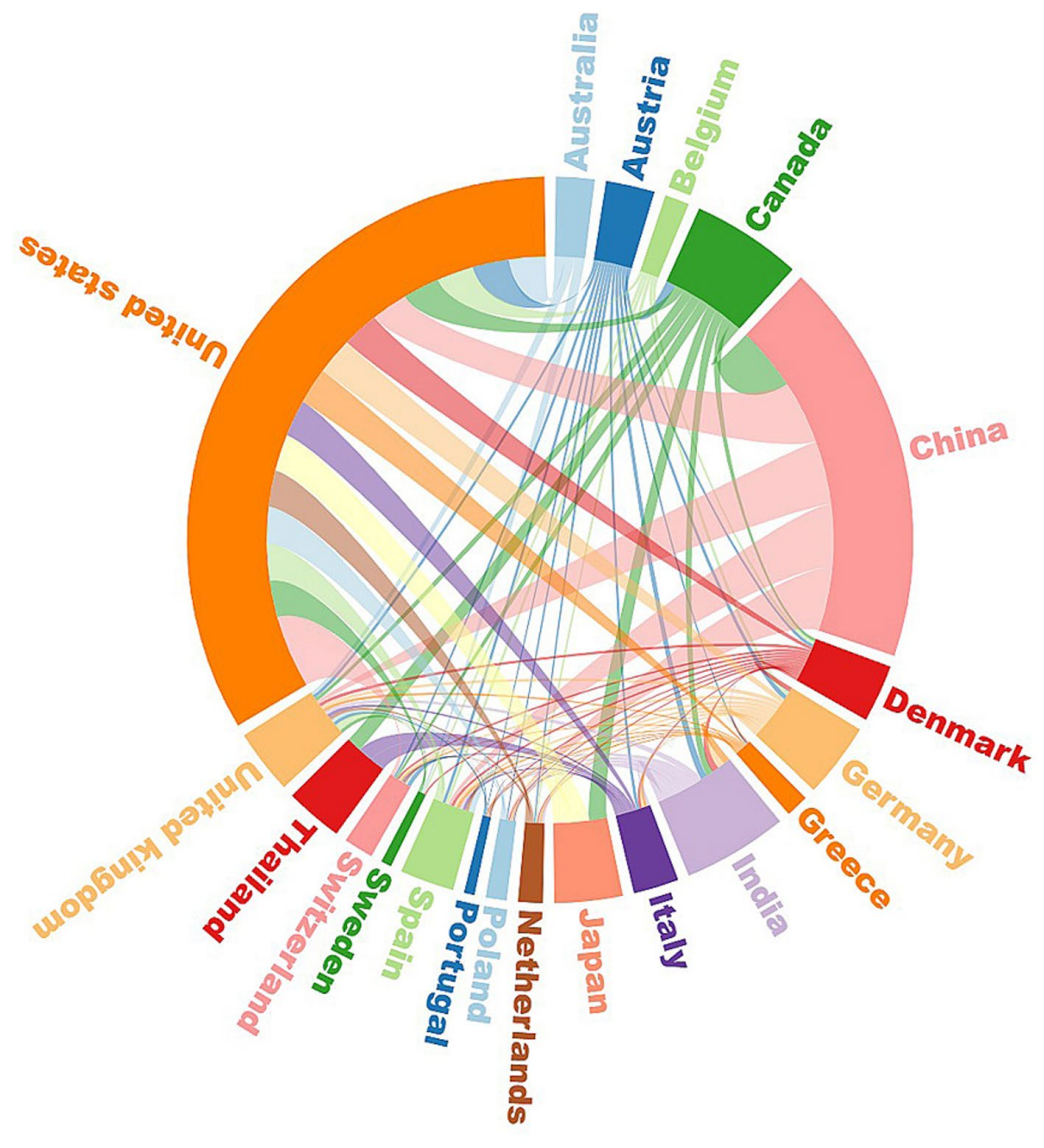
International collaboration network among countries. The chord diagram displays the 20 most productive countries/regions using full counting. Each country/region was counted once per article, and multinational articles generated pairwise collaboration links. Segment size represents publication output, whereas ribbon width represents the number of jointly authored articles between each pair of countries/regions.

The country/region collaboration map (Figure 4) showed broad international participation. The United States contributed the largest publication volume and had visible collaborative links with China, the United Kingdom, and Canada. China ranked second in publication output. These patterns may partly reflect database coverage, English-language restriction, and discipline-specific publication practices.

The institutional analysis (Figure 3B) identified the Hospital for Special Surgery as the most productive institution. Stanford University, Mayo Clinic, and the University of Toronto were also visible network hubs. Their centrality reflects publication and collaboration patterns in the indexed dataset and should not be interpreted as a direct measure of institutional research quality.

### 3.4 Analysis of references and keywords

#### 3.4.1 Keyword clustering

**Figure 5:**
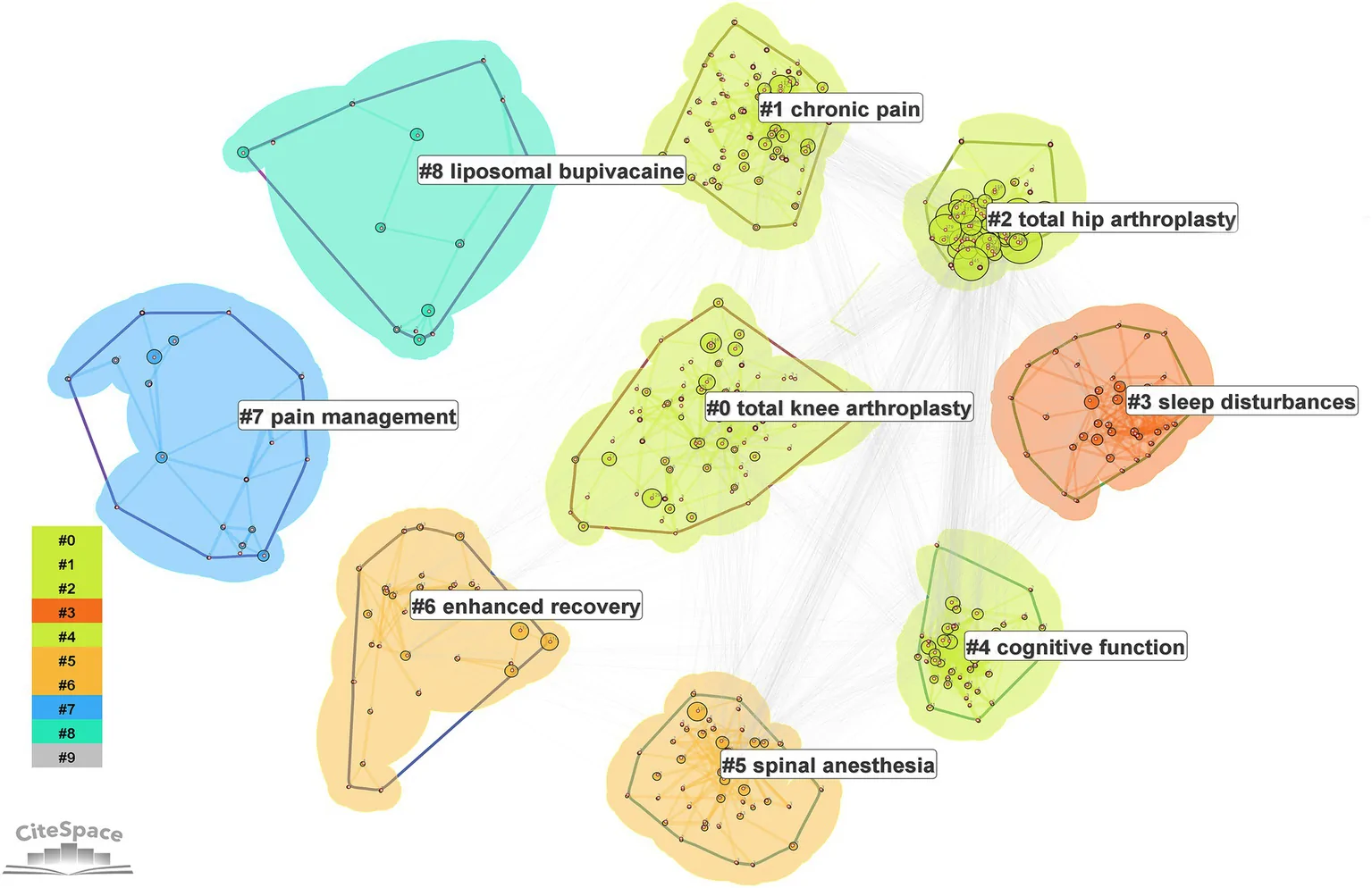
Keyword co-occurrence clustering map of research on peripheral nerve blocks for total knee arthroplasty, 2016–2025. The CiteSpace analysis identified nine major keyword clusters, including total knee arthroplasty (#0), chronic pain (#1), total hip arthroplasty (#2), sleep disturbances (#3), cognitive function (#4), spinal anesthesia (#5), enhanced recovery (#6), pain management (#7), and liposomal bupivacaine (#8). Node size is proportional to keyword occurrence frequency, and links represent co-occurrence relationships between keywords. Colored regions and cluster labels indicate the thematic groups identified by CiteSpace. The clustering structure showed satisfactory quality, with a modularity *Q* value >0.7 and a mean silhouette value >0.8. Cluster presence and size reflect thematic concentration within the literature rather than clinical priority or evidence strength.

CiteSpace keyword clustering identified nine clusters, with satisfactory structural quality (modularity *Q* > 0.7 and mean silhouette >0.8; Figure 5). The most frequent keywords were total knee arthroplasty (*n* = 821), nerve blocks (*n* = 464), analgesia (*n* = 408), postoperative pain (*n* = 379), and femoral nerve block (*n* = 331) (Table 1).

**Table 1:** Top 20 most frequently occurring keywords in peripheral nerve blocks for total knee arthroplasty (2016–2025).

Rank	Keywords	Frequency, *n*	Rank	Keywords	Frequency, *n*
1	Total knee arthroplasty	821	11	Double blind procedure	164
2	Nerve blocks	464	12	Local infiltration analgesia	161
3	Analgesia	408	13	Pain management	156
4	Postoperative pain	379	14	Arthroplasty	150
5	Femoral nerve block	331	15	Arthroplasty, replacement, knee	150
6	Adductor canal block	311	16	Regional anesthesia	146
7	Pain	270	17	Bupivacaine	144
8	Postoperative analgesia	199	18	Efficacy	143
9	Surgery	181	19	Replacement	133
10	Ropivacaine	165	20	Major clinical study	132

The principal clusters encompassed total knee arthroplasty, total hip arthroplasty, enhanced recovery, liposomal bupivacaine, spinal anesthesia, chronic pain, sleep disturbance, and cognitive function, reflecting the major thematic domains represented in the dataset. The identification of sleep- and cognition-related clusters indicates that these outcomes were present in the literature, although the clustering analysis alone does not establish a temporal shift in research priorities.

#### 3.4.2 Analysis of co-citation bursts

**Figure 6:**
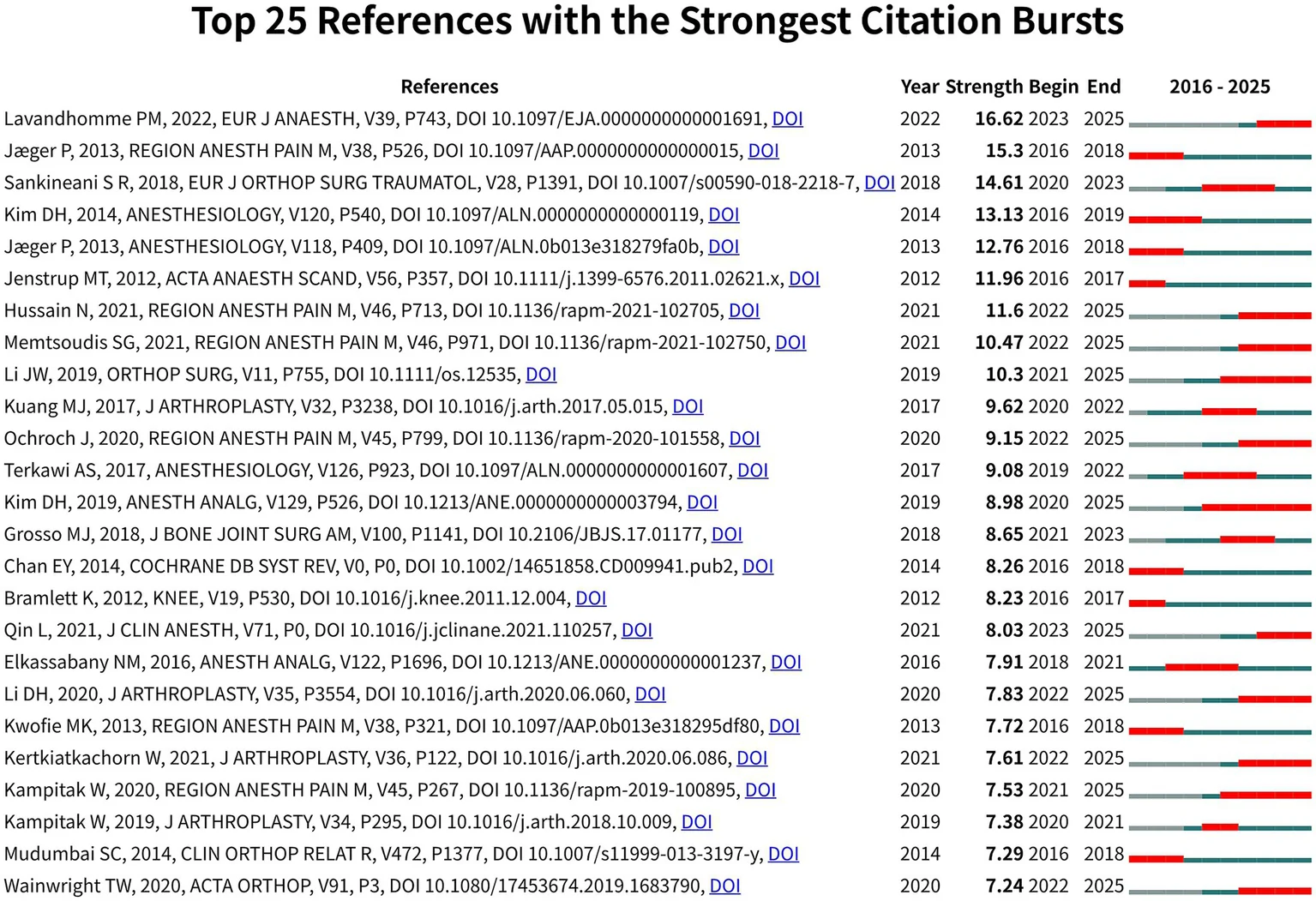
Top 25 references with the strongest citation bursts in research on peripheral nerve blocks for total knee arthroplasty, 2016–2025. References are ranked by burst strength. “Year” indicates the publication year, “Strength” represents the intensity of the citation burst, and “Begin” and “End” indicate the starting and ending years of the burst period. The horizontal timeline covers 2016–2025, with red segments denoting periods during which a reference experienced a marked increase in citation activity. Citation bursts identify periods of rapidly increasing scholarly attention and should not be interpreted as direct indicators of methodological quality or clinical importance.

The 25 references with the strongest citation bursts are presented in Figure 6. Earlier bursts included studies associated with the growing visibility of ACB as a motor-sparing alternative to FNB, whereas more recent bursts involved procedure-specific recommendations and systematic reviews addressing refined motor-sparing approaches (6, 10–12). These burst patterns identify references that received a marked increase in scholarly attention during specific periods.

## 4 Discussion

### 4.1 Interpreting the bibliometric patterns in the context of TKA analgesia

The bibliometric maps demonstrate sustained research interest in PNBs for TKA and their close association with multimodal analgesia and enhanced recovery after surgery (ERAS). The co-occurrence of PNB-related terms with ERAS, opioid-sparing care, and functional recovery suggests that contemporary research increasingly considers analgesia within broader perioperative recovery pathways. This pattern is consistent with procedure-specific recommendations and evidence syntheses supporting opioid- and motor-sparing strategies in selected clinical settings (10, 13–15).

Several recurrent themes—including ACB, IPACK, chronic postsurgical pain, and functional outcomes—reflect continuing efforts to improve analgesic coverage while preserving motor function and supporting recovery. Persistent pain remains an important outcome after TKA (4, 5, 16), which may explain the visibility of chronic pain–related terms in the thematic structure. However, the presence of these themes primarily reflects the questions receiving sustained research attention and does not, by itself, establish the effectiveness of any specific block strategy in preventing chronic postsurgical pain.

The substantial publication output from China indicates rapid growth in research activity and an increasing contribution to the global literature. Translating this academic activity into clinical practice will nevertheless depend on locally relevant evidence regarding training, resources, safety, comparative effectiveness, and cost. Similarly, the prominence of liposomal bupivacaine reflects sustained scientific and clinical interest, including ongoing debate regarding its incremental value over conventional local anesthetic regimens (17–20).

### 4.2 Motor-sparing techniques as a prominent research theme

Citation-burst and keyword analyses indicate the evolving prominence of motor-sparing techniques, with ACB and IPACK becoming increasingly visible alongside the earlier emphasis on FNB. This thematic pattern reflects growing interest in balancing analgesia with preservation of quadriceps strength, early mobilization, and functional recovery.

Randomized trials and meta-analyses comparing ACB with FNB have reported better preservation of motor function in some settings, while analgesic outcomes have generally been comparable (6, 7, 21, 22). These findings provide a clinical context for the increasing prominence of ACB within the bibliometric network, although outcomes may vary according to block technique, local anesthetic regimen, background multimodal analgesia, and rehabilitation protocol.

IPACK has also become a frequently investigated technique because it is intended to provide posterior knee analgesia while limiting motor blockade. Systematic reviews evaluating the addition of IPACK to ACB have reported heterogeneous findings across studies, partly because of differences in background analgesia, block protocols, and outcome definitions (12, 23, 24). Its bibliometric prominence therefore reflects continued investigation of its potential role within multimodal and ERAS pathways rather than a settled consensus regarding its incremental clinical benefit.

### 4.3 Pharmacological innovation: the liposomal bupivacaine debate

Liposomal bupivacaine formed a distinct keyword cluster, indicating sustained interest in extended-duration local anesthetic formulations. Its visibility spans both periarticular infiltration and peripheral nerve block applications, reflecting efforts to prolong postoperative analgesia and reduce reliance on opioids or continuous catheter techniques (25, 26).

However, the clinical literature remains inconsistent regarding its advantages over conventional local anesthetic regimens. The persistence of this theme may therefore reflect a combination of therapeutic interest, commercial visibility, repeated comparative investigation, uncertainty regarding clinical benefit, and concerns about cost-effectiveness. Liposomal bupivacaine should consequently be regarded as an active and debated research topic rather than an established superior strategy.

### 4.4 Patient-centered outcomes as represented research themes

The presence of sleep disturbance, cognitive function, and other recovery-related outcomes in the thematic network suggests that the literature extends beyond immediate postoperative pain intensity and opioid consumption. These outcomes are clinically relevant because sleep disruption, neurocognitive symptoms, pain, opioid exposure, and rehabilitation may interact during recovery after TKA (27, 28).

Their representation in the network supports growing interest in broader patient-centered recovery domains. Nevertheless, cluster presence alone is insufficient to demonstrate a definitive temporal shift from pain-centered research toward multidimensional recovery assessment; such a conclusion would require overlay visualization, thematic-evolution analysis, or time-stratified keyword mapping.

Current clinical evidence provides a rationale for further investigation of sleep and cognitive outcomes, but it remains uncertain whether individual PNB techniques directly improve these domains. Future longitudinal and comparative studies should therefore incorporate standardized patient-reported outcomes, sleep measures, neurocognitive assessments, opioid exposure, and functional recovery endpoints.

### 4.5 Research gaps suggested by the bibliometric map

Several research gaps can be formulated from the thematic and collaboration patterns. First, the visibility of motor-sparing techniques supports comparative studies using standardized definitions of block technique, background multimodal analgesia, and clinically meaningful outcomes, including mobility, falls, discharge readiness, adverse events, and longer-term recovery.

Second, the prominence and continued citation activity surrounding liposomal bupivacaine highlight the need for adequately powered comparative-effectiveness and health-economic studies. Such studies should distinguish periarticular infiltration from peripheral nerve block administration, use transparent comparators, and account for institutional pricing and resource differences. This is a research priority arising from uncertainty and publication activity, not a recommendation for or against routine clinical use.

Third, the concentration of influential authors and institutions within several collaboration clusters suggests opportunities for broader multicenter and cross-regional studies. Greater participation from underrepresented regions and harmonization of outcome definitions could improve generalizability. The bibliometric analysis does not identify a preferred PNB combination for ERAS; treatment selection should rely on current clinical guidelines, comparative evidence, patient characteristics, and local expertise.

Future research should extend follow-up beyond the immediate postoperative period and include validated patient-reported outcomes, functional recovery milestones, sleep quality, cognitive outcomes, and chronic postsurgical pain. Longitudinal bibliometric analyses should also test whether these topics are increasing over time rather than inferring temporal change from cluster presence alone. These directions define an evidence-generation agenda and should not be read as direct clinical recommendations (29, 30).

## 5 Limitations

This study has several limitations that should be considered when interpreting the findings.

A principal limitation concerns database coverage. The analysis was restricted to the WoSCC and Scopus. Although combining these databases broadened bibliographic coverage, differences in journal selection, citation indexing, subject classification, and keyword fields may have influenced publication trends, citation rankings, geographic and institutional representation, collaboration networks, and thematic patterns. The exclusion of PubMed/MEDLINE, Embase, Dimensions, and regional databases further limits the comprehensiveness of the dataset. The findings should therefore be regarded as a broad, but not exhaustive, representation of the field.

The language and document-type restrictions represent another potential source of selection bias. Only English-language documents classified as Articles were included. Relevant research published in other languages or disseminated through conference proceedings, editorials, letters, and other forms of scholarly communication may therefore have been underrepresented, particularly in regions where research is less frequently published in English-language journals.

Citation-based indicators are subject to several well-recognized limitations. Citation counts are influenced by publication age, journal visibility, database coverage, language, geographic publication patterns, discipline-specific citation practices, and self-citation. Older articles have had more time to accumulate citations, whereas recent studies may receive fewer citations despite addressing emerging topics. To ensure comparability, WoSCC Times Cited values were used as the prespecified source for database-derived citation indicators and publication rankings. Scopus-only records were retained for publication, authorship, collaboration, and keyword analyses but were excluded from indicators requiring directly comparable citation counts; citation counts from the two databases were neither summed nor averaged. Author self-citations were retained, and no sensitivity analysis excluding self-citations was performed because sufficiently reliable matching of author identities across all citing and cited records could not be ensured. Citation counts should therefore be interpreted primarily as indicators of database-recorded scholarly visibility. Citation-burst results are likewise influenced by database coverage and publication timing and represent periods of increased scholarly attention rather than evidence quality or clinical importance.

The integration of records from two databases introduced additional challenges related to field mapping, hierarchical deduplication, author-name disambiguation, and institutional normalization. Despite automated procedures and manual review, residual errors may remain because of common author names, name variants, affiliation changes, institutional mergers, abbreviations, and alternative English translations. In the institutional analysis, affiliated hospitals were assigned to their formally affiliated parent universities. Although this approach reduced institutional fragmentation, it may have increased the apparent contribution of universities with multiple affiliated hospitals and obscured hospital-level differences.

Finally, keyword co-occurrence, clustering, and citation-burst analyses characterize thematic prominence and changes in scholarly attention but do not evaluate the methodological quality, risk of bias, consistency, or certainty of the underlying clinical evidence. A prominent theme may reflect clinical relevance, controversy, commercial visibility, methodological uncertainty, or repeated investigation without consensus. Furthermore, the study was based primarily on bibliographic metadata and did not include a formal thematic-evolution analysis or systematic appraisal of full-text clinical evidence. The findings therefore cannot establish the clinical superiority of a specific intervention or confirm a definitive temporal shift in research priorities and should be considered complementary to systematic reviews, meta-analyses, health-economic evaluations, and clinical guidelines.

## 6 Conclusion

This bibliometric analysis characterized publication activity, collaboration networks, citation structures, and thematic development in articles on PNBs for TKA published between 2016 and 2025. Within the databases examined, the United States and China were the leading contributors, while several major academic institutions occupied central positions in the collaboration networks. Motor-sparing approaches, particularly ACB and IPACK, together with liposomal bupivacaine, chronic postsurgical pain, and broader recovery-related outcomes, emerged as prominent research themes. These patterns highlight areas of sustained scholarly interest and opportunities for broader international and multicenter collaboration.

By providing a transparent and reproducible overview of the intellectual and collaborative structure of the field, this study identifies underexplored areas and priorities for future research. The observed bibliometric prominence reflects publication and scholarly attention rather than evidence of the clinical superiority of any specific analgesic strategy; clinical decisions should continue to be informed by comparative evidence, guideline recommendations, patient characteristics, and local resources.

## Data Availability

The author-generated reproducibility materials supporting this study have been deposited in Zenodo and are publicly available at https://doi.org/10.5281/zenodo.21508542. The repository includes the final cleaned derived bibliographic dataset comprising 1,225 retained records, separate derived metadata tables for WoSCC and Scopus records, screening and deduplication logs, a data dictionary, keyword and country/region normalization materials, software and network-analysis settings, validation and quality-control documentation, the complete database-specific search strategies, and the reconstructed PNBTKAScreen R workflow. Because the original preprocessing involved both automated procedures and manual adjudication, the deposited code is described as a reconstructed reproducibility workflow rather than the original contemporaneous execution code. The general merged dataset does not contain the final institutional-name harmonization used for the institution-level analyses. The institution-normalized dataset and its corresponding thesaurus are maintained separately and are available from the corresponding authors upon reasonable request. Unmodified bibliographic exports obtained directly from the Web of Science Core Collection and Scopus are not publicly redistributed because access and reuse are governed by database subscription and licensing conditions. Researchers with appropriate database access can reproduce the retrieval process using the complete search strategies, filters, retrieval date, eligibility criteria, and extraction protocol provided in the Zenodo repository. Original database exports may be supplied confidentially to the editorial office where permitted by database and journal policies.
