# Supplementary material for: Peripheral nerve blocks for total knee arthroplasty: a bibliometric and visual analysis, 2016–2025
Source: Front Med (Lausanne). 2026 Aug 17;13:1903949. doi: 10.3389/fmed.2026.1903949 (PMC13526949; doi:10.3389/fmed.2026.1903949)
Supplement: Supplementary file 2 [file Table_1.doc]

**Supplementary Methods and Reproducibility Materials**

# S1. Database-specific search strategies

| **Database** | **Exact search date** | **Collection/index** | **Field** | **Exact query** | **Applied filters** | **Export format** |
| --- | --- | --- | --- | --- | --- | --- |
| WoSCC | January 7, 2026 | Web of Science Core Collection | TS (Topic) | TS=(("total knee arthroplasty" OR TKA OR "total knee replacement") AND ("peripheral nerve block*" OR "adductor canal block*" OR "femoral nerve block*" OR IPACK OR iPACK OR "local infiltration analgesia")) AND PY=(2016-2025) AND LA=(English) AND DT=(Article) | Publication years 2016–2025; English; Article only | Plain text; full record and cited references |
| Scopus | January 7, 2026 | Scopus | TITLE-ABS-KEY | TITLE-ABS-KEY(("total knee arthroplasty" OR TKA OR "total knee replacement") AND ("peripheral nerve block*" OR "adductor canal block*" OR "femoral nerve block*" OR IPACK OR iPACK OR "local infiltration analgesia")) AND PUBYEAR > 2015 AND PUBYEAR < 2026 AND LIMIT-TO(LANGUAGE,"English") AND LIMIT-TO(DOCTYPE,"ar") | Publication years 2016–2025; English; article (ar) | CSV; citation information and bibliographical information |

# S2. Eligibility and screening rules

| **Domain** | **Include** | **Exclude** |
| --- | --- | --- |
| Population/procedure | TKA or total knee replacement; a separately identifiable TKA subgroup | Non-TKA surgery without separately identifiable TKA data |
| Intervention/topic | Evaluation, comparison, or description of at least one named peripheral nerve block | Systemic analgesia, neuraxial anesthesia, or local infiltration alone without a named peripheral nerve block |
| Document type | Article | Review, conference paper/proceeding, meeting abstract, editorial, letter, note, correction, book chapter, and other non-article publication types |
| Language | English | Non-English |
| Publication year | 2016–2025 | Outside 2016–2025 |
| Local infiltration analgesia | Retained only when a named peripheral nerve block was evaluated in the same study or comparative framework | Periarticular, intra-articular, or wound infiltration alone without a nerve-targeted injection or named peripheral nerve block |
| Publication status | Early-access, online-first, or article-in-press records classified as Articles were eligible. When both a preliminary and final version existed, the records were consolidated and the final publication metadata were retained. A unique preliminary record without a matching final version was retained as one publication. | The preliminary version was not retained as a separate record when a matching final version was identified. |

Screening was performed independently by two investigators using titles, abstracts, document types, and available metadata. Full text was consulted when eligibility could not be determined from metadata alone. Disagreements were resolved through discussion and consensus.

# S3. Data integration, deduplication, and citation-data rules

**1. DOI normalization.** DOI strings were converted to lowercase; URL prefixes (https://doi.org/ and http://doi.org/), the prefix doi:, embedded spaces, and terminal punctuation were removed.

**2. Exact DOI matching.** Records with identical normalized DOIs were grouped as duplicate candidates.

**3. Exact normalized-title matching.** For records with missing or discordant DOI information, titles were normalized using Unicode normalization, lowercase conversion, punctuation removal, whitespace standardization, and removal of non-informative formatting. Identical normalized titles were flagged as duplicate candidates.

**4. Metadata-assisted matching.** Unresolved records were compared using normalized title, first author, publication year, and journal or source title. Minor discrepancies were referred for manual review rather than being merged automatically.

**5. Fuzzy title matching.** Potential title variants were assessed using duplicatedMatching in the bibliometrix R package (Field = "TI", exact = FALSE, tol = 0.95), which applies a restricted Damerau–Levenshtein similarity measure. The prespecified similarity threshold was 0.95.

**6. Manual adjudication.** Candidate duplicate pairs were independently reviewed by two investigators using DOI, title, author list, publication year, journal, volume, issue, pages or article number, abstract, and other available metadata. Disagreements were resolved by consensus. Records remained separate when duplicate status was uncertain.

**7. Master-record selection.** For confirmed cross-database duplicates, the WoSCC record was retained as the master record. A Scopus record was retained when the publication was uniquely retrieved from Scopus. Non-conflicting fields could be supplemented from the corresponding duplicate; conflicting fields followed the selected master source.

**8. Missing data.** Missing metadata were retained as missing and were not imputed.

**9. Citation-data harmonization and self-citations.** WoSCC Times Cited was the prespecified source for database-derived citation indicators and publication rankings. Scopus-only records were retained for publication, authorship, institution, country/region, collaboration, and keyword analyses but excluded from indicators requiring a directly comparable citation count. WoSCC and Scopus citation counts were neither summed nor averaged. Author self-citations were retained, and no self-citation-exclusion sensitivity analysis was performed because reliable identity matching across all citing and cited records could not be ensured.

**10. Final integrated dataset.** The combined 924 WoSCC and 899 Scopus records yielded 1,823 pre-deduplication records. After removal of 598 duplicates, the final integrated dataset comprised 1,225 retained publications: 922 WoSCC master records and 303 Scopus-only records.

# S4. Metadata normalization and analytical parameters

## S4.1 Metadata normalization and entity disambiguation

| **Entity** | **Procedure** | **Conservative decision rule / counting consequence** |
| --- | --- | --- |
| Authors | Automated normalization of punctuation, spacing, capitalization, initials, and name order, followed by manual review using full names, ORCID identifiers, affiliations, publication years, research topics, and recurring co-author patterns. | Author entities were merged only when multiple metadata elements supported a common identity. Uncertain entities remained separate. |
| Institutions | Prespecified institutional thesaurus covering abbreviations, alternative English translations, department variants, historical names, mergers, and hospital–university relationships. | Affiliated hospitals were assigned to formally affiliated parent universities; independent hospitals remained separate. This may increase parent-university counts and obscure hospital-level differences. |
| Countries/regions | Alternative names and database-specific variants were converted to preferred standardized forms. | Each distinct country/region was counted once per article; multinational articles credited every participating country/region and generated pairwise collaboration links. |
| Keywords | Prespecified keyword thesaurus harmonizing capitalization, spelling, British/American English, hyphenation, singular/plural forms, abbreviations, and closely equivalent terms. | Original and preferred forms are documented in the deposited keyword thesaurus. |
| Citation-count source | WoSCC Times Cited for database-derived citation indicators and rankings. | Scopus-only records were excluded from indicators requiring comparable publication-level citation counts; counts were not combined across databases. |

## S4.2 Software and network-analysis settings

| **Analysis / software** | **Settings** | **Included entities / interpretation** |
| --- | --- | --- |
| R / bibliometrix | R version 4.1.2; bibliometrix used for annual output, authorship, collaboration indicators, citation summaries, and fuzzy title matching. | CAGR calculated as [(N2025/N2016)^(1/9) − 1] × 100; 3-year moving average descriptive only. |
| Author co-authorship / VOSviewer 1.6.20 | Full counting; association-strength normalization; minimum 2 publications; no citation threshold. | 188 authors. Node size = publication output; link width = co-authorship strength; color = cluster membership. |
| Institutional co-authorship / VOSviewer 1.6.20 | Full counting; association-strength normalization; minimum 3 publications; institution-normalized data. | 86 institutions. All distinct normalized affiliations retained; affiliated hospitals assigned to parent universities. |
| Country/region collaboration | Full counting; minimum 5 publications for network eligibility; the chord diagram displays the 20 most productive countries/regions. | Each country counted once per article. Segment size = publication output; ribbon width = bilateral co-authorship count. |
| Journal co-citation / VOSviewer 1.6.20 | Minimum 5 citations; association-strength normalization. | 65 journals. Node size = citation frequency; link width = co-citation strength. |
| Keyword clustering / CiteSpace 6.2.R4 | Time span 2016–2025; 1-year slices; log-likelihood-ratio labels; minimum cluster size 20 nodes. | Nine clusters; modularity Q > 0.7 and mean silhouette > 0.8. |
| Reference citation bursts / CiteSpace 6.2.R4 | Time span 2016–2025; burst detection applied to cited references. | Top 25 strongest bursts displayed; burst periods represent rapidly increasing scholarly attention. |
| Visualization readability | VOSviewer label-overlap reduction; enlarged exports for dense networks. | Visualization settings affected label display only, not node inclusion, weights, links, or cluster assignments. |

# S5. Flow-diagram reconciliation table

| **Stage** | **WoSCC** | **Scopus** | **Combined/unique** | **Excluded at this stage** |
| --- | --- | --- | --- | --- |
| Records identified | 1,289 | 1,401 | – | – |
| Non-English records excluded | 17 | 80 | – | 97 |
| English-language records retained | 1,272 | 1,321 | – | – |
| Non-article document types excluded | 348 | 422 | – | 770 |
| Articles retained before merging | 924 | 899 | – | – |
| Records merged | – | – | 1,823 | – |
| Duplicate records removed | – | – | – | 598 |
| Unique records after deduplication | – | – | 1,225 | – |
| Local-infiltration-only records excluded after deduplication | – | – | – | 0 |
| Other irrelevant records excluded after deduplication | – | – | – | 0 |
| Final articles included | – | – | 1,225 | – |

# S6. Reproducibility repository and access conditions

The author-generated reproducibility materials have been deposited in Zenodo and are publicly available at <https://doi.org/10.5281/zenodo.21508542>. The record includes the final cleaned derived metadata table containing 1,225 retained records, separate derived WoSCC and Scopus metadata tables, screening and deduplication logs, a data dictionary, keyword and country/region normalization materials, software and network-analysis settings, quality-control documentation, the complete database-specific search and extraction protocol, and the reconstructed PNBTKAScreen R workflow.

Because the original preprocessing involved automated procedures and manual adjudication, the deposited code is described as a reconstructed reproducibility workflow rather than the original contemporaneous execution code. The institution-normalized dataset and its corresponding thesaurus used for institution-level analyses are maintained separately and are available from the corresponding authors upon reasonable request.

Unmodified WoSCC and Scopus exports are not publicly redistributed because their access and reuse are governed by database subscription and licensing conditions. Researchers with appropriate database access can reconstruct the retrieval process using the exact queries, filters, retrieval date, eligibility criteria, matching parameters, and processing rules reported here and in the repository.
